# Supplementary material for: The gut microbiota composition is linked to subsequent occurrence of ventilator-associated pneumonia in critically ill patients
Source: Microbiol Spectr. 2023 Sep 15;11(5):e00641-23. doi: 10.1128/spectrum.00641-23 (PMC10581192; doi:10.1128/spectrum.00641-23)
Supplement: Supplemental Materials — Supplemental tables, figures, and script. [file spectrum.00641-23-s0001.docx]

**Supplemental Table 1.** Microbiologic isolates from ventilator-associated pneumonia patients

| **Bacterial species isolated on pulmonary samples from patients with ventilator-associated pneumonia (n: 18)** | |
| --- | --- |
| Polymicrobial pneumonia | 7 (39%) |
| *Citrobacter koseri* | 4 (22%) |
| *Klebsiella pneumoniae* | 3 (17%) |
| *Klebsiella varicola* | 3 (17%) |
| *Escherichia coli* | 6 (14%) |
| *Pseudomonas aeruginosa* | 2 (11%) |
| *Staphylococcus aureus* | 2 (11%) |
| *Serratia marcescens* | 2 (11%) |
| *Enterococcus faecalis* | 2 (11%) |
| *Enterococcus faecium* | 1 (5%) |
| *Acinetobacter baumanii* | 1 (5%) |
| *Haemophilus influenza* | 1 (5%) |

**Supplemental Table 2.** ANCOM-BC differential taxonomy analysis for gut bacteriobiota.

Reference for analysis: no_VAP group.

|  | **coefficients** | **Standard errors** | **Test statistics** | **P-Values** | **Adjusted p-values** |
| --- | --- | --- | --- | --- | --- |
| *Mobiluncus curtisii* | -0.1502801 | 0.4601480 | -0.3265909 | <0.0001 | <0.0001 |
| *Varibaculum cambriense* | -0.3518085 | 0.3645142 | -0.9651436 | <0.0001 | <0.0001 |
| *Corynebacterium aurimucosum* | -0.2608604 | 0.4343736 | -0.6005438 | <0.0001 | <0.0001 |
| *Corynebacterium pyruviciproducens* | 0.1323093 | 0.4973873 | 0.2660087 | <0.0001 | <0.0001 |
| *Rothia mucilaginosa* | -0.1332012 | 0.3652258 | -0.3647091 | <0.0001 | <0.0001 |
| *Cutibacterium avidum* | -0.0832586 | 0.3745106 | -0.2223130 | <0.0001 | <0.0001 |
| *Gordonibacter pamelaeae* | 0.7009735 | 0.3575474 | 1.9605052 | <0.0001 | <0.0001 |
| *Bacteroides salyersiae* | 0.9423533 | 0.5535057 | 1.7025179 | <0.0001 | <0.0001 |
| *Coprobacter secundus* | -0.3378530 | 0.2928328 | -1.1537403 | <0.0001 | <0.0001 |
| *Porphyromonas asaccharolytica* | -0.4925282 | 0.6456726 | -0.7628141 | <0.0001 | <0.0001 |
| *Porphyromonas somerae* | 0.4007976 | 0.5529030 | 0.7248968 | <0.0001 | <0.0001 |
| *Prevotella timonensis* | -0.5293592 | 0.5634441 | -0.9395061 | <0.0001 | <0.0001 |
| *Alistipes inops* | 1.1447785 | 0.5944942 | 1.9256345 | <0.0001 | <0.0001 |
| *Campylobacter ureolyticus* | -0.3769149 | 0.5165202 | -0.7297195 | <0.0001 | <0.0001 |
| *Dielma fastidiosa* | -0.2080099 | 0.5037891 | -0.4128908 | <0.0001 | <0.0001 |
| *Faecalicoccus pleomorphus* | -0.2366629 | 0.4887508 | -0.4842199 | <0.0001 | <0.0001 |
| *Holdemania filiformis* | -0.2254425 | 0.3136304 | -0.7188158 | <0.0001 | <0.0001 |
| *Facklamia hominis* | 0.0726721 | 0.5305590 | 0.1369727 | <0.0001 | <0.0001 |
| *Streptococcus mutans* | 0.2033487 | 0.4448152 | 0.4571532 | <0.0001 | <0.0001 |
| *Lachnoclostridium edouardi* | -0.7196894 | 0.4882070 | -1.4741481 | <0.0001 | <0.0001 |
| *Lachnospiraceae_NK4A136_group bacterium* | -0.3506030 | 0.5331647 | -0.6575885 | <0.0001 | <0.0001 |
| *Colidextribacter massiliensis* | 0.0583271 | 0.3803308 | 0.1533588 | <0.0001 | <0.0001 |
| *Negativibacillus massiliensis* | 0.2906513 | 0.3751770 | 0.7747044 | <0.0001 | <0.0001 |
| *Ruminococcus champanellensis* | -0.5094219 | 0.5251998 | -0.9699582 | <0.0001 | <0.0001 |
| *Peptococcus niger* | -0.3445985 | 0.4159488 | -0.8284638 | <0.0001 | <0.0001 |
| *Acidaminococcus fermentans* | -0.5321886 | 0.6726090 | -0.7912303 | <0.0001 | <0.0001 |
| *Acidaminococcus intestini* | -0.2397403 | 0.7460424 | -0.3213494 | <0.0001 | <0.0001 |
| *Phascolarctobacterium succinatutens* | -0.5753684 | 0.6921723 | -0.8312502 | <0.0001 | <0.0001 |
| *Dialister invisus* | -0.6218365 | 0.4693492 | -1.3248908 | <0.0001 | <0.0001 |
| *Megasphaera massiliensis* | -0.9291615 | 0.5558382 | -1.6716401 | <0.0001 | <0.0001 |
| *Negativicoccus succinicivorans* | -0.4544560 | 0.2981850 | -1.5240739 | <0.0001 | <0.0001 |
| *Veillonella parvula* | -0.3468579 | 0.5194075 | -0.6677954 | <0.0001 | <0.0001 |
| *Cloacibacillus porcorum* | 0.6281372 | 0.6765234 | 0.9284782 | <0.0001 | <0.0001 |
| *Pyramidobacter piscolens* | -0.0269926 | 0.5022644 | -0.0537417 | <0.0001 | <0.0001 |
| *Victivallis vadensis* | -0.1435288 | 0.3038198 | -0.4724141 | <0.0001 | <0.0001 |

**Supplemental Table 3.** ANCOM-BC differential taxonomy analysis for gut mycobiota

Reference for analysis: no_VAP group.

|  | **coefficients** | **Standard errors** | **Test statistics** | **P-Values** | **Adjusted p-values** |
| --- | --- | --- | --- | --- | --- |
| *Cladosporium sp.* | -0.0879899 | 0.6456186 | -0.1362878 | <0.0001 | <0.0001 |
| *Alternaria sp.* | 2.2082751 | 0.8239358 | 2.6801544 | <0.0001 | <0.0001 |
| *Clavispora sp.* | -0.3654562 | 0.3694097 | -0.9892978 | <0.0001 | <0.0001 |
| *Nakaseomyces sp.* | 1.7680072 | 1.4599369 | 1.2110162 | <0.0001 | <0.0001 |
| *Rhodotorula sp.* | -0.5062703 | 0.5293344 | -0.9564281 | <0.0001 | <0.0001 |

**Supplemental Figure 1.** Non metric Bray-curtis analysis of β-diversity of the V3-V4 sequencing run. Mock: mock community. TN: negative control from extraction step. TN PCR: negative control from amplification PCR step.


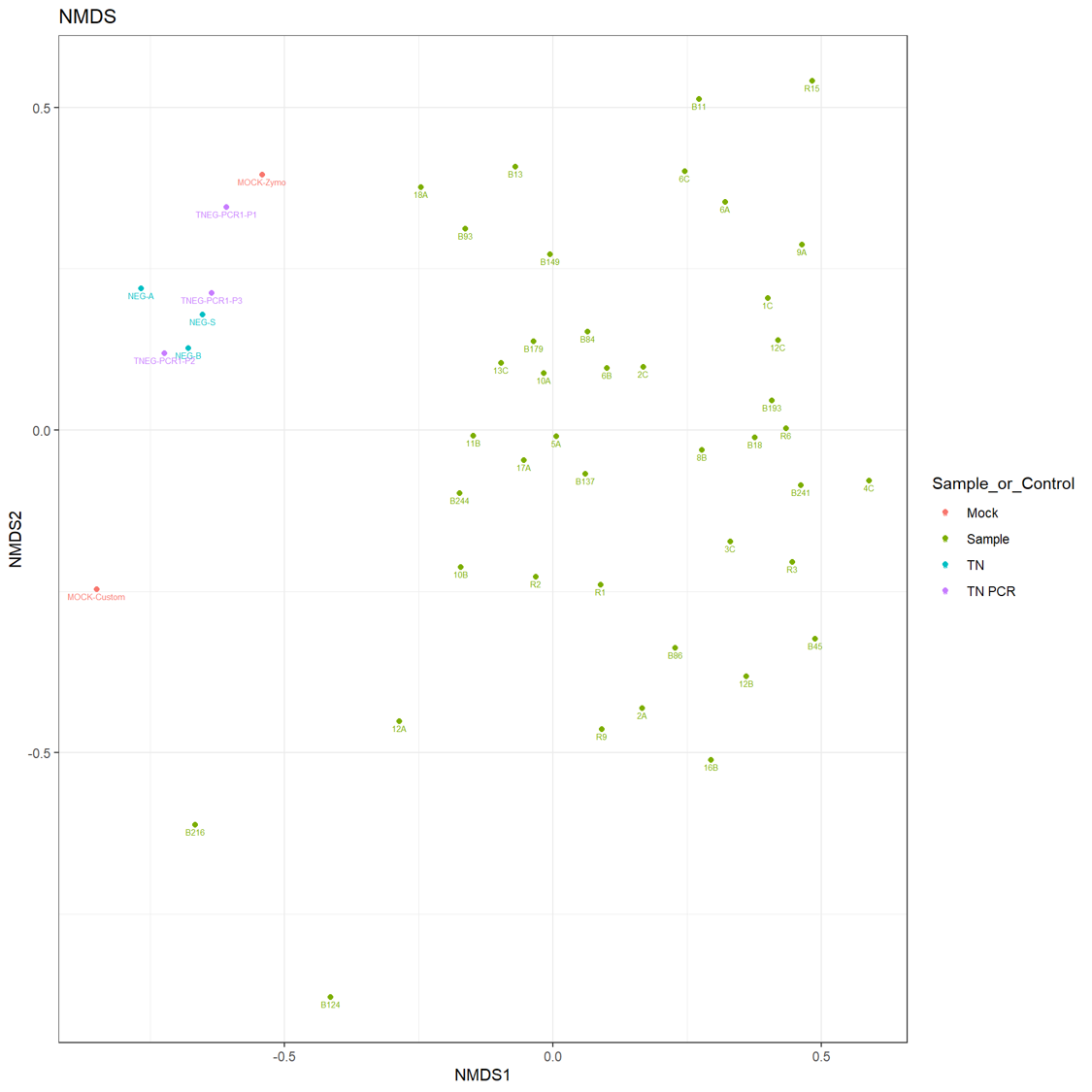


**Supplemental Figure 2.** Non metric Bray-curtis analysis of β-diversity of the ITS2 sequencing run. Mock: mock community. TN: negative control from extraction step. TN PCR: negative control from amplification PCR step.


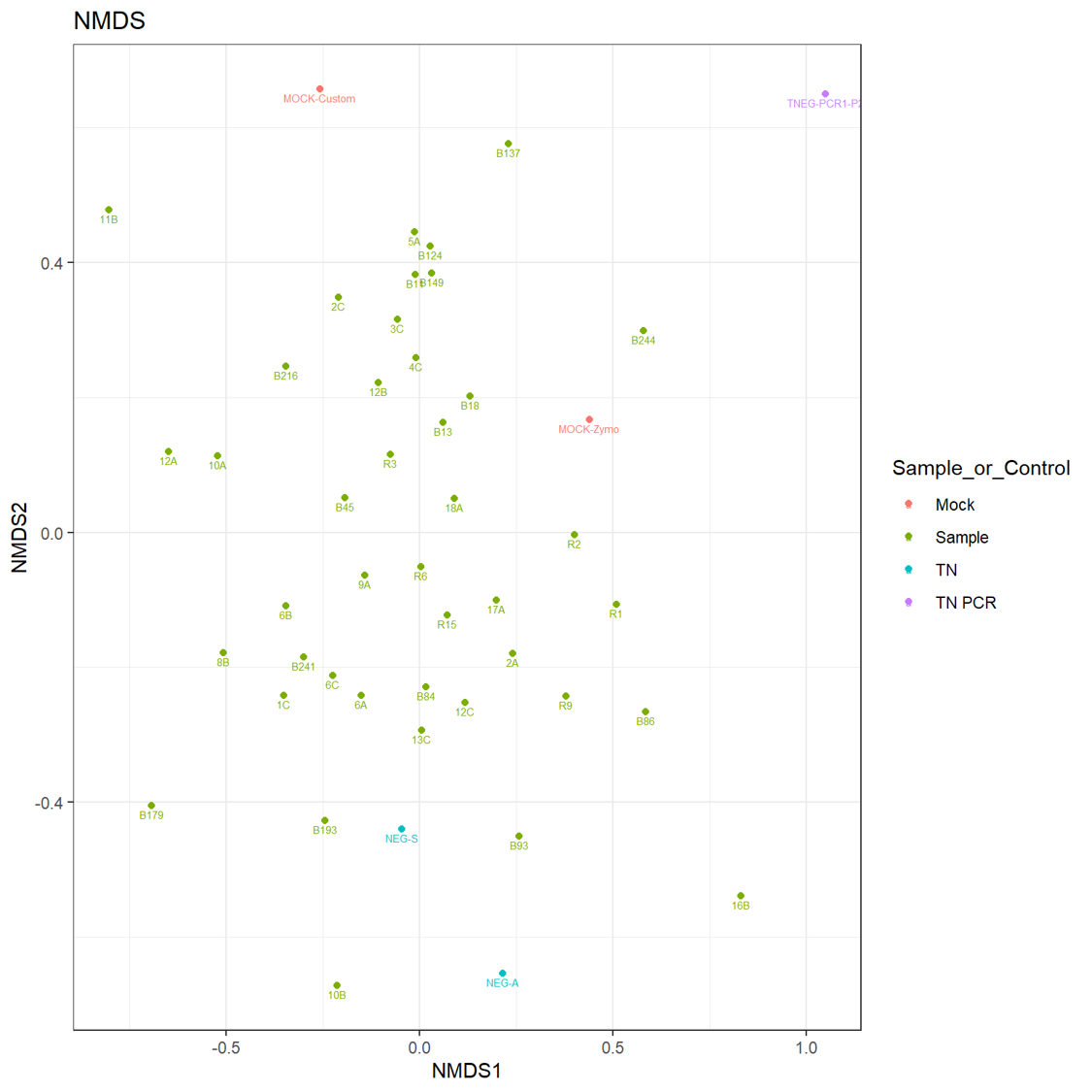


**Supplemental Figure 3**. Comparison of gut bacteriobiota between patients who received antimicrobial therapy within the past 3 months before admission and those who did not. A. Boxplot of estimated α-diversity by Shannon index. B. Boxplot of estimated α-diversity by Simpson index. C. Boxplot of estimated α-diversity by evenness. D. Metric Bray-curtis analysis of β-diversity. Larger filled circles indicate group centroids. Ellipses indicate the 95% confidence interval around the centroid in non-dimensional space. Threshold for statistical significance: p=0.05. atb: antibiotics.


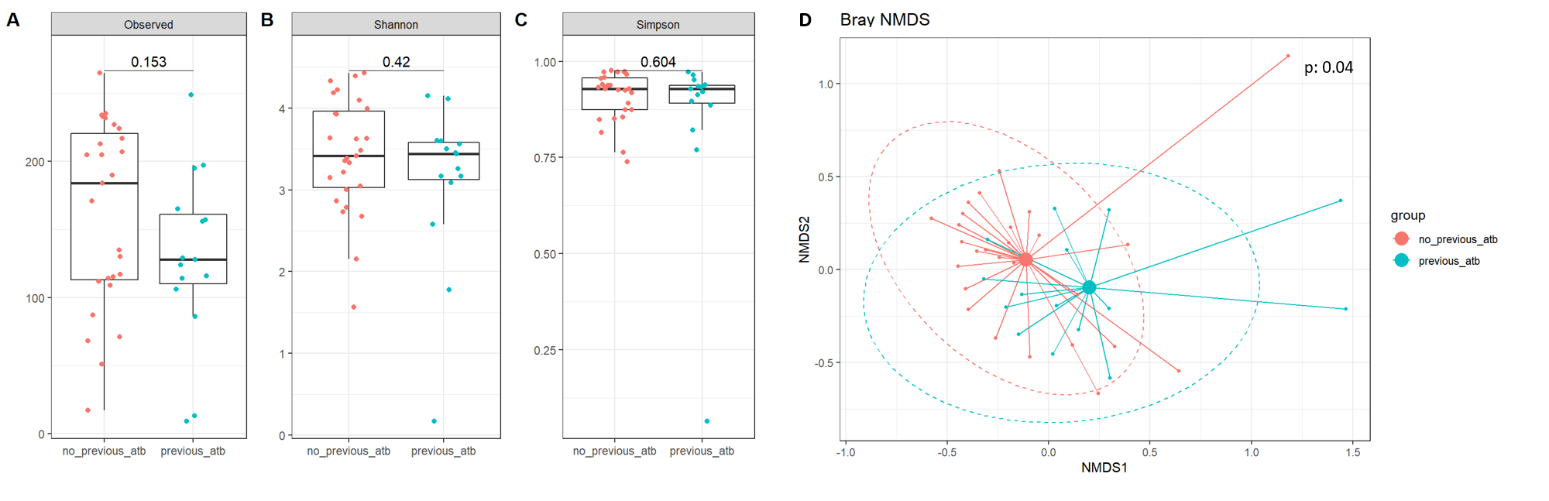


**Supplemental Figure 4**. Comparison of gut mycobiota between patients who received antimicrobial therapy within the past 3 months before admission and those who did not. A. Boxplot of estimated α-diversity by Shannon index. B. Boxplot of estimated α-diversity by Simpson index. C. Boxplot of estimated α-diversity by evenness. D. Metric Bray-curtis analysis of β-diversity. Larger filled circles indicate group centroids. Ellipses indicate the 95% confidence interval around the centroid in non-dimensional space. Threshold for statistical significance: p=0.05. atb: antibiotics.


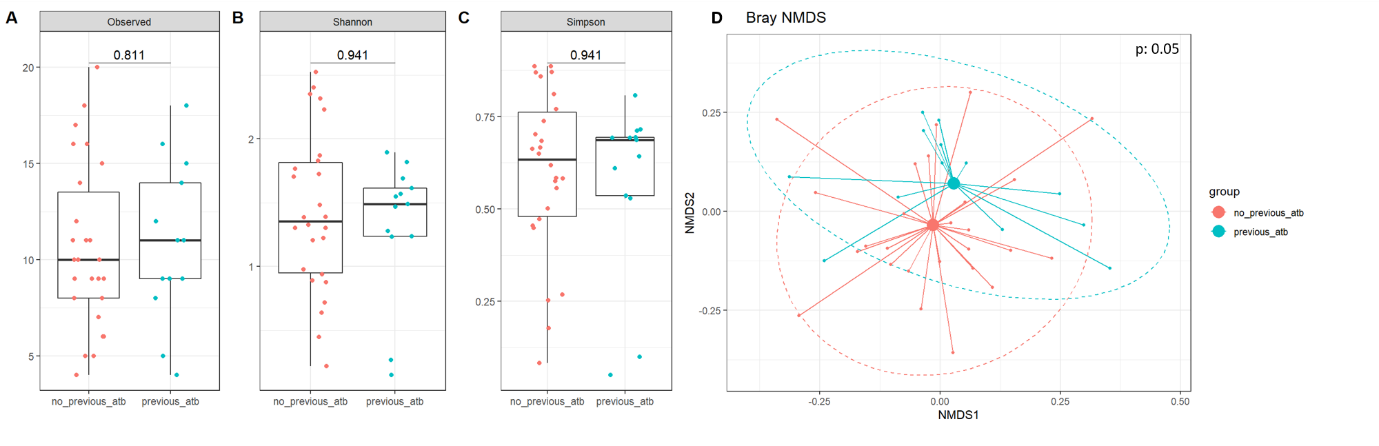


**Supplemental Figure 5**. Normalized reads abundance of A. the bacterial species and B. the fungal genus associated with the occurrence of ventilator-associated pneumonia. VAP= ventilator-associated pneumonia.

**
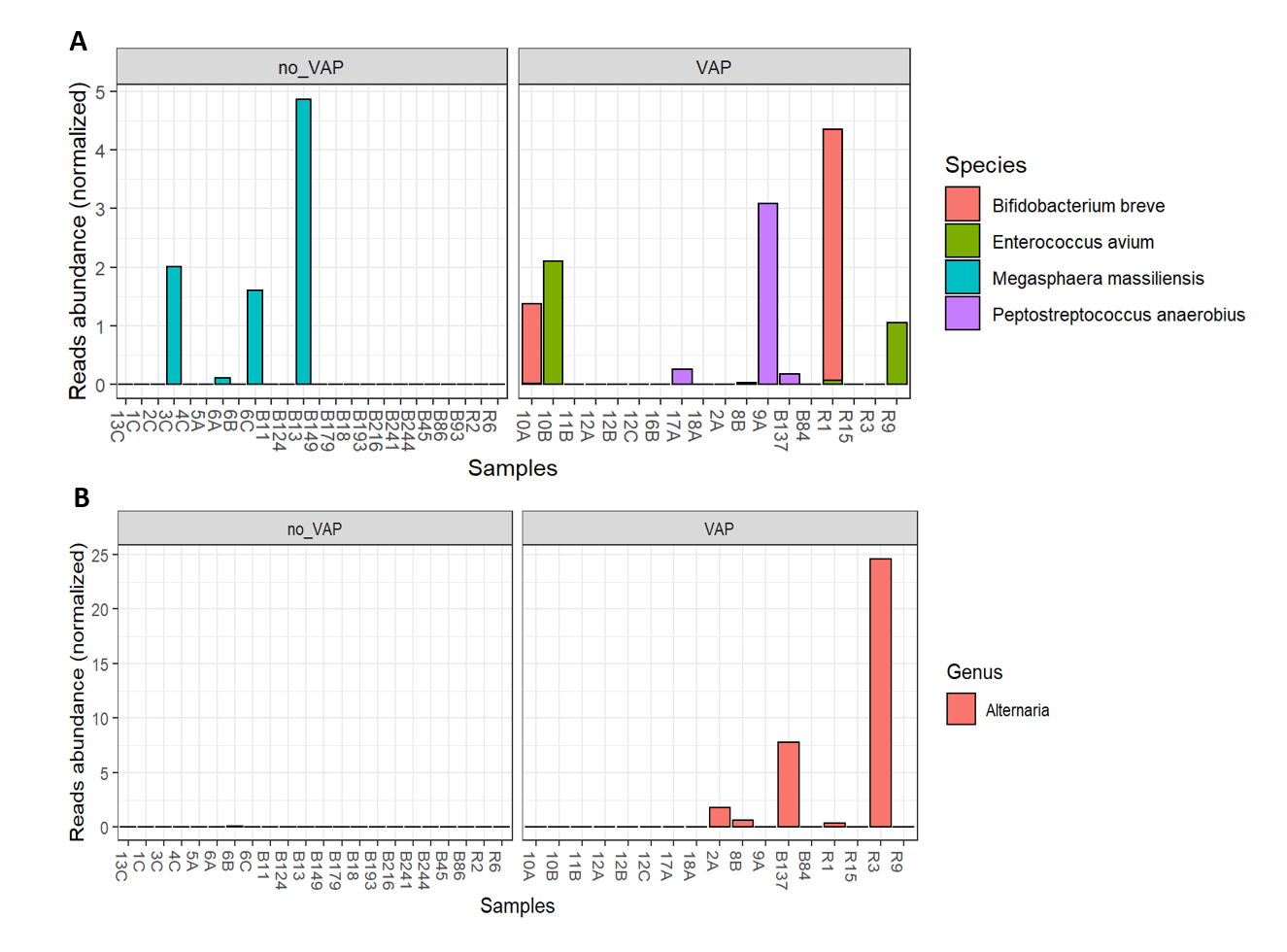
**

**Mock**

**ZymoBIOMICS® Microbial Community Standard**

*Listeria monocytogenes* - 12%

*Pseudomonas aeruginosa* - 12%

*Bacillus subtilis* - 12%

*Escherichia coli* - 12%

*Salmonella enterica* - 12%

*Lactobacillus fermentum* - 12%

*Enterococcus faecalis* - 12%

*Staphylococcus aureus* - 12%

*Saccharomyces cerevisiae* - 2%

*Cryptococcus neoformans* - 2%

**r16SRNA coding gene home-made mock microbial community standards**

*Streptococcus mitis* (400 µL)

*Streptococcus oralis* (400 µL)

*Pseudomonas aeruginosa* (200 µL)

*Stenotrophomonas maltophilia* (200 µL)

*Staphylococcus epidermidis* (400 µL)

*Staphylococcus aureus* (400 µL)

*Acinetobacter baumannii* (400 µL)

*Klebsiella pneumoniae* (200 µL)

*Proteus mirabilis* (200 µL)

*Serratia marcescens* (400 µL)

*Lactobacillus spp.* (200 µL)

*Escherichia coli* (ATCC 25922) (200 µL)

*Enterobacter cloacae* (200µL)

*Enterococcus faecalis* (ATCC 29212) (400 µL)

**ITS2 coding gene home-made mock microbial community standards**

*Scedosporium apiospermum* (*Pseudallescheria boydii*) (200 µL)

*Mucor circillenoides* (400 µL)

*Candida lusitaniae* (*Clavispora lusitaniae*) (400 µL)

*Scedosporium aurantiacum* (400 µL)

*Lomentospora prolificans* (*Scedosporium prolificans*) (200 µL)

*Aspergillus fumigatus* (400 µL)

*Aspergillus flavus* (200 µL)

*Aspergillus terreus* (200 µL)

*Penicillium griseofulvum* (200 µL)

*Fusarium solani* (200 µL)

*Candida dubliniensis* (400 µL)

*Candida albicans* (ATCC 5314) (400 µL)

*Rhodotorula mucilaginosa* (400 µL)

*Exophiala dermatitidis* (400 µL)

**Script for 16S RNA coding gene assignation**

---

title: "Assignation 16S"

author: "Renaud Prevel & Raphael Enaud"

date: '`r format(Sys.time(), "%d %m %Y")`'

header-includes:

- \usepackage{color, fancyvrb}

output:

rmdformats::readthedown:

highlight: kate

numbersections : yes

html_document:

fig_height: 7

fig_width: 10

---

```{r knitrinit, include=FALSE, warning=FALSE, message=FALSE, cache=FALSE}

library(rstudioapi)

setwd(dirname(rstudioapi::getActiveDocumentContext()$path))

load("./assignation.rda")

library(knitr)

library(rmdformats)

library("kableExtra")

library(DESeq2)

library("ggplot2")

library("readxl")

library("dplyr")

library(knitr)

library(rmdformats)

library(psy)

library(Rcpp)

library(nnet)

library(dplyr)

library(ggplot2)

library(psy)

library(prettyR)

library(corrplot)

library(readr)

library(questionr)

library(finalfit)

library(labelled)

library("dada2")

library(ShortRead)

library(Biostrings)

library(DECIPHER)

library("plyr")

theme_set(theme_bw())

library (phyloseq)

library(BiocStyle)

library(ape)

library(tidyr)

library(broom)

library(dplyr)

library(Hmisc)

library (microbiome)

library (picante)

library(plyr)

library(reshape2)

library(doBy)

library(RVAideMemoire)

library (vegan)

library(heatmap.plus)

library(RColorBrewer)

library(gplots)

library(kableExtra)

library(gridExtra)

library(knitr)

library(microbiomeSeq)

library(adespatial)

library(ggpubr)

library (devtools)

library(yingtools2)

library("microDecon")

```

```{r, include=FALSE}

# Creation of the phyloseq object

samdf<- read_xlsx("./Sample Data.xlsx")

samples_df <- sample_data(samdf)

rownames(samples_df) <- samples_df$sample

samples_df$Groupe <- factor(samples_df$Groupe)

samples_df$Sample_or_Control <- factor(samples_df$Sample_or_Control)

phylo <- phyloseq(otu_table(seqtab.nochim, taxa_are_rows=FALSE),

sample_data(samples_df),

tax_table(taxa))

dna <- Biostrings::DNAStringSet(taxa_names(phylo))

names(dna) <- taxa_names(phylo)

phylo <- merge_phyloseq(phylo, dna)

taxa_names(phylo) <- paste0("ASV", seq(ntaxa(phylo)))

phylo

```

# Check the number of reads

```{r, echo=FALSE}

df <- as.data.frame(sample_data(phylo)) # Put sample_data into a ggplot-friendly data.frame

df$LibrarySize <- sample_sums(phylo)

df <- df[order(df$LibrarySize),]

df$Index <- seq(nrow(df))

ggplot(data=df, aes(x=Index, y=LibrarySize, color=Sample_or_Control, label = sample)) + geom_point()

kableExtra::kable(sort(sample_sums(phylo))) %>%

kable_styling() %>%

scroll_box(width = "100%", height = "600px")

```

# Selection of bacterial ASVs

```{r, echo=FALSE}

phylo = subset_taxa(phylo, Kingdom=="Bacteria")

phylo

#Removal of phylum with a single taxa

phylo <- subset_taxa(phylo, !is.na(Phylum) & !Phylum %in% c("","Bdellovibrionota","Chloroflexi","Deinococcota","<NA>","NA"))

phylo

```

# Filtering of minority ASVs

```{r, echo=FALSE}

# ASV present in less than 3 samples

condition <- function(x) { sum(x > 0) >= 2 }

taxaToKeep <- filter_taxa(phylo, condition)

phylo <- prune_taxa(taxaToKeep, phylo)

phylo = prune_samples(names(which(sample_sums(phylo) >= 1)), phylo)

phylo = prune_taxa(names(which(taxa_sums(phylo) >= 1)), phylo)

phylo

```

# Rarefaction curves

```{r, include=FALSE, warning=FALSE, message=FALSE, cache=FALSE, fig.width = 10, fig.height= 10}

library(vegan)

library(ggplot2)

library("ranacapa")

phylo = prune_samples(names(which(sample_sums(phylo) >= 1)), phylo)

rarefactioncurves = ggrare(phylo, step = 100, label = "sample", color = "Sample_or_Control",se = FALSE)

```

```{r, echo=FALSE, warning=FALSE, message=FALSE, cache=FALSE, fig.width = 10, fig.height= 10}

rarefactioncurves

```

# Beta diversity

Measurement of Beta Diversity by Bray Curits

## MDS representation (multi-dimensional scaling)

```{r, include=FALSE, warning=FALSE, message=FALSE, cache=FALSE, fig.width = 10, fig.height= 10}

# Transform data to proportions as appropriate for Bray-Curtis distances

ps.prop <- transform_sample_counts(phylo, function(otu) otu/sum(otu))

ord.nmds.bray <- ordinate(ps.prop, method="MDS", distance="bray")

```

```{r, echo=FALSE, warning=FALSE, message=FALSE, cache=FALSE, fig.width = 10, fig.height= 10}

plot_ordination(ps.prop, ord.nmds.bray, type="samples", label = "sample", color = "Sample_or_Control", title="MDS")

```

## Representation NMDS (Non-metric multi-dimensional scaling)

```{r, include=FALSE, warning=FALSE, message=FALSE, cache=FALSE, fig.width = 10, fig.height= 10}

ord.nmds.bray <- ordinate(ps.prop, method="NMDS", distance="bray")

```

```{r, echo=FALSE, warning=FALSE, message=FALSE, cache=FALSE, fig.width = 10, fig.height= 10}

plot_ordination(ps.prop, ord.nmds.bray, type="samples", label = "sample", color = "Sample_or_Control",title="NMDS")

```

# Beta diversity tree (with Bray Curtis dissimilarity index)

```{r, include=FALSE, warning=FALSE, message=FALSE, cache=FALSE}

library("ape")

random_tree = rtree(ntaxa(phylo), rooted=TRUE, tip.label=taxa_names(phylo))

phylo1 = merge_phyloseq(phylo,random_tree)

phylo1

# Dissimilarity measurement with Bray Cutris

GPUF <- round(distance(phylo1,"bray"),3)

```

```{r, echo=FALSE, warning=FALSE, message=FALSE, cache=FALSE, fig.width = 7, fig.height= 26}

# Creating a tree

## Manually define color-shading vector based on sample type.

colorScale <- rainbow(length(levels(getVariable(phylo1, "Sample_or_Control"))))

cols <- colorScale[getVariable(phylo1, "Sample_or_Control")]

GP.tip.labels <- as(getVariable(phylo1, "sample"), "character")

GP.hclust <- hclust(GPUF, method = "average")

plot(as.phylo(GP.hclust), show.tip.label = TRUE, tip.color = "white")

tiplabels(GP.tip.labels, col = cols, frame = "none",adj = -0.05,cex = 0.7)

```

# Removal of negative controls and Mock

```{r, echo=FALSE, warning=FALSE, message=FALSE, cache=FALSE}

phylo <- subset_samples(phylo, Sample_or_Control =="sample")

phylo = prune_taxa(names(which(taxa_sums(phylo) >= 1)), phylo)

phylo

```

# Resume of the final dataset

```{r, echo=FALSE, warning=FALSE, message=FALSE, cache=FALSE}

summarize_phyloseq(phylo)

```

# saving the global environment

```{r, echo=FALSE, warning=FALSE, message=FALSE, cache=FALSE}

save(phylo, list,file="Phylo pre processed.rda")

```

**Script for ITS2 coding gene assignation**

---

title: "ITS Assignment"

author: "Renaud Prevel & Raphael Enaud"

date: '`r format(Sys.time(), "%d %m %Y")`'

header-includes:

- \usepackage{color, fancyvrb}

output:

rmdformats::readthedown:

highlight: kate

numbersections : yes

html_document:

fig_height: 7

fig_width: 10

---

```{r knitrinit, include=FALSE, warning=FALSE, message=FALSE, cache=TRUE}

# In "path", create a folder "Fastq" with the sequences R1 and R2.

library(knitr)

library(rmdformats)

library("kableExtra")

library(DESeq2)

library("ggplot2")

library("readxl")

library("dplyr")

library(knitr)

library(rmdformats)

library("kableExtra")

library(DESeq2)

library(psy)

library(Rcpp)

library(nnet)

library(dplyr)

library(ggplot2)

library(psy)

library(prettyR)

library(corrplot)

library(readr)

library(questionr)

library(finalfit)

library(labelled)

library("dada2")

library(ShortRead)

library(Biostrings)

library(DECIPHER)

library("plyr")

library("phyloseq")

library("ranacapa")

theme_set(theme_bw())

library(rstudioapi)

setwd(dirname(rstudioapi::getActiveDocumentContext()$path))

path <- file.path(".")

input <-list.dirs(path, full.names = F, recursive = F)

qual <-"./quality/"

outp <-"./output/"

fastq <- "./Fastq"

R1 <- "./R1"

fnFs <- sort(list.files(fastq, pattern = "_R1_001.fastq.gz", full.names = TRUE))

fnRs <- sort(list.files(fastq, pattern = "_R2_001.fastq.gz", full.names = TRUE))

## ITS2 Primers (ITS7F / 3271R):

FWD <- "GTGARTCATCGAATCTTT"

REV <- "GATATGCTTAAGTTCAGCGGGT"

```

```{r, include=FALSE, warning=FALSE, message=FALSE, cache=FALSE}

# Checking the presence and orientation of primers

allOrients <- function(primer) {

# Create all orientations of the input sequence

require(Biostrings)

dna <- DNAString(primer) # The Biostrings works w/ DNAString objects rather than character vectors

orients <- c(Forward = dna, Complement = Biostrings::complement(dna), Reverse = reverse(dna),

RevComp = reverseComplement(dna))

return(sapply(orients, toString)) # Convert back to character vector

}

FWD.orients <- allOrients(FWD)

REV.orients <- allOrients(REV)

FWD.orients

REV.orients

# Pre filtration

fnFs.filtN <- file.path(fastq, "filtN", basename(fnFs)) # Put N-filterd files in filtN/ subdirectory

fnRs.filtN <- file.path(fastq, "filtN", basename(fnRs))

out <- filterAndTrim(fnFs, fnFs.filtN, fnRs, fnRs.filtN, maxN = 0, multithread = TRUE)

primerHits <- function(primer, fn) {

# Counts number of reads in which the primer is found

nhits <- vcountPattern(primer, sread(readFastq(fn)), fixed = FALSE)

return(sum(nhits > 0))

}

```

Number of primers found :

```{r, echo=FALSE, warning=FALSE, message=FALSE, cache=FALSE}

rbind(FWD.ForwardReads = sapply(FWD.orients, primerHits, fn = fnFs.filtN[[1]]),

FWD.ReverseReads = sapply(FWD.orients, primerHits, fn = fnRs.filtN[[1]]),

REV.ForwardReads = sapply(REV.orients, primerHits, fn = fnFs.filtN[[1]]),

REV.ReverseReads = sapply(REV.orients, primerHits, fn = fnRs.filtN[[1]]))

```

# Primer removal

```{r, include=FALSE, warning=FALSE, message=FALSE, cache=FALSE}

cutadapt <- "C:/Users/EnaudR/AppData/Local/Packages/PythonSoftwareFoundation.Python.3.9_qbz5n2kfra8p0/LocalCache/local-packages/Python39/Scripts/cutadapt.exe" # CHANGE ME to location on your machine

R1 <- file.path(path, "R1")

if(!dir.exists(R1)) dir.create(R1)

R2 <- file.path(path, "R2")

if(!dir.exists(R2)) dir.create(R2)

fnFs.cut <- file.path(R1, basename(fnFs))

fnRs.cut <- file.path(R2, basename(fnRs))

FWD.RC <- dada2:::rc(FWD)

REV.RC <- dada2:::rc(REV)

# Trim FWD and the reverse-complement of REV off of R1 (forward reads)

R1.flags <- paste("-g", FWD, "-a", REV.RC)

# Trim REV and the reverse-complement of FWD off of R2 (reverse reads)

R2.flags <- paste("-G", REV, "-A", FWD.RC)

# Run Cutadapt

for(i in seq_along(fnFs)) {

system2(cutadapt, args = c(R1.flags, R2.flags, "-n", 2, # -n 2 required to remove FWD and REV from reads

"-m", 20, "-o", fnFs.cut[i], "-p", fnRs.cut[i], # output files, filtrage des reads au minimum 20 pb

fnFs.filtN[i], fnRs.filtN[i])) # input files

}

```

```{r, echo=FALSE, warning=FALSE, message=FALSE, cache=FALSE}

rbind(FWD.ForwardReads = sapply(FWD.orients, primerHits, fn = fnFs.cut[[1]]),

FWD.ReverseReads = sapply(FWD.orients, primerHits, fn = fnRs.cut[[1]]),

REV.ForwardReads = sapply(REV.orients, primerHits, fn = fnFs.cut[[1]]),

REV.ReverseReads = sapply(REV.orients, primerHits, fn = fnRs.cut[[1]]))

```

```{r, include=FALSE, warning=FALSE, message=FALSE, cache=FALSE}

path <- file.path(".")

input<-list.dirs(path, full.names = F, recursive = F)

qual<-"./quality/"

outp<-"./output/"

R1 = "./R1"

write.fasta.dada<-function(dada2, file){

seqs<-dada2::getSequences(dada2)

hash<-paste0(">",sapply(seqs, openssl::sha1, USE.NAMES = F))

write(c(rbind(hash, seqs)),file)

}

for (R1 in R1){

# List FASTQ files

fq <-list.files(file.path(path,"R1"), pattern="_001.fastq.gz")

# Extract sample names, assuming filenames have format: SAMPLENAME_XXX.fastq

sample.names <- sapply(strsplit(fq, "_"), `[`, 1)

# Add the full path

fq <- file.path(R1, fq)

filt_path <- file.path(qual,"R1") # Place filtered files in subdirectory

dir.create(filt_path, recursive = T)

# Prepare future filenames

filt <- file.path(filt_path, paste0(sample.names, ".filt.fastq.gz"))

out.ee <- ldply(1:length(sample.names),function(i) {

fastqFilter(fn = fq[i],

fout = filt[i],

trimLeft = 0,minLen = 100,

maxN=0, maxEE = 1, rm.phix=TRUE,

compress=TRUE)

})

# Dereplication

derep <- llply(1:length(sample.names), function(i){

derepFastq(filt[i], verbose=TRUE)

})

names(derep)<-sample.names

# Learn errors

err <- learnErrors(derep, multithread=3)

# Get variants

dada_obj<-dada(derep, err=err, multithread = 3)

# Create output directory

#outp_rds<-file.path(outp,R1, "RDS")

#dir.create(outp_rds, recursive = T)

#saveRDS(dada_obj, file = paste0(outp_rds,"/",R1,".RDS"), compress = "gzip")

# Produce Variant Table

seqtab<-makeSequenceTable(dada_obj)

# Remove Chimeras

seqtab.nochim <- removeBimeraDenovo(seqtab, verbose=TRUE)

}

```

# List of samples

```{r, echo=FALSE, warning=FALSE, message=FALSE, cache=FALSE}

sample.names

```

# Checking reading quality profiles

```{r, echo=FALSE, warning=FALSE, message=FALSE, cache=FALSE}

plotQualityProfile(fq[1:2])

```

# Filtering reads

```{r, echo=FALSE, warning=FALSE, message=FALSE, cache=FALSE}

# Place filtered files in filtered/ subdirectory

```

# Learning about error rates

```{r, echo=FALSE, warning=FALSE, message=FALSE, cache=FALSE}

plotErrors(err, nominalQ=TRUE)

```

# Bulding an ASVs table

```{r, echo=FALSE, warning=FALSE, message=FALSE, cache=FALSE}

seqtab <- makeSequenceTable(dada_obj)

dim(seqtab)

```

# Checking the distribution of sequence lengths

```{r, echo=FALSE, warning=FALSE, message=FALSE, cache=FALSE}

options(max.print="500")

table(nchar(getSequences(seqtab)))

```

# Remove chimeras

```{r, echo=FALSE, warning=FALSE, message=FALSE, cache=FALSE}

seqtab.nochim <- removeBimeraDenovo(seqtab, verbose=TRUE)

dim(seqtab.nochim)

sum(seqtab.nochim)/sum(seqtab)

table(nchar(getSequences(seqtab.nochim)))

```

# Summary of the different steps of the pipeline

```{r, include=FALSE, warning=FALSE, message=FALSE, cache=FALSE}

getN <- function(x) sum(getUniques(x))

track <- cbind(out, sapply(dada_obj, getN), rowSums(seqtab.nochim))

## If processing a single sample, remove the sapply calls: e.g. replace sapply(dadaFs, getN) with getN(dadaFs)

colnames(track) <- c("input", "filtered", "denoised", "nonchim")

rownames(track) <- sample.names

```

```{r, echo=FALSE, warning=FALSE, message=FALSE, cache=FALSE}

options(max.print="600")

track

```

# Assignment

```{r, include=FALSE, warning=FALSE, message=FALSE, cache=FALSE}

library(stringr)

unite.ref <- "./sh_general_release_dynamic_s_10.05.2021.fasta" # CHANGE ME to location on your machine

taxa <- assignTaxonomy(seqtab.nochim, unite.ref, multithread = TRUE, tryRC = TRUE)

str_replace_all(taxa, pattern = "k__", replacement = "")

str_replace_all(taxa, pattern = "p__", replacement = "")

str_replace_all(taxa, pattern = "c__", replacement = "")

str_replace_all(taxa, pattern = "o__", replacement = "")

str_replace_all(taxa, pattern = "f__", replacement = "")

str_replace_all(taxa, pattern = "g__", replacement = "")

str_replace_all(taxa, pattern = "s__", replacement = "")

```

Viewing the found assignments :

```{r, echo=FALSE, warning=FALSE, message=FALSE, cache=FALSE}

taxa.print <- taxa # Removing sequence rownames for display only

rownames(taxa.print) <- NULL

head(taxa.print)

```

# saving the global environment

save( out.ee, fq, seqtab, seqtab.nochim, err, track,sample.names, taxa , file = "./assignation Unite.rda")

```
